# Inhibition of Hydrogen Evolution by a Bifunctional Membrane between Anode and Electrolyte of Aluminum–Air Battery

**DOI:** 10.3390/membranes12040407

**Published:** 2022-04-06

**Authors:** Yuxin Zuo, Ying Yu, Haoqin Shi, Jiale Wang, Chuncheng Zuo, Xiaowei Dong

**Affiliations:** 1 College of Fashion Design, Jiaxing Nanhu University, Jiaxing 314000, China; yuxinzuo@zjxu.edu.cn; 2 College of Information Science and Engineering, Jiaxing University, Jiaxing 314000, China; Vickydon@zjxu.edu.cn; 3 School of Mechanical Engineering & Automation, Zhejiang Sci-Tec University, Hangzhou 310018, China; 202030605242@mails.zstu.edu.cn (H.S.); 201930506056@mails.zstu.edu.cn (J.W.)

**Keywords:** bifunctional membrane, Al_2_O_3_@PAN, hydrogen evolution reaction, anti-corrosion, Al–air battery

## Abstract

The hydrogen evolution reaction of the anode is a severe barrier that limits the further commercial application of Al–air batteries. Therefore, this study introduces a bifunctional membrane for the inhibition of hydrogen evolution in Al–air batteries. The reference to Al_2_O_3_@PAN as “bifunctional” means that it has both hydrophobic and anti-corrosion functions. Al_2_O_3_ can effectively inhibit the migration of hydroxide ions, and PAN is an excellent hydrophobic material. The bifunctional membrane is placed between the aluminum anode and the electrolyte, which can prevent the invasion of excess water and hydroxide ions, thereby inhibiting the hydrogen evolution corrosion of the anode. Electrochemical tests have confirmed that the corrosion inhibition rate of a bifunctional membrane containing 1.82 wt. % Al_2_O_3_@PAN is as high as 89.24%. The specific capacity of Al–air batteries containing this membrane can reach 1950 mAh/g, and the utilization rate of the aluminum anode has reached 61.2%, which is helpful in reducing the waste of aluminum resources. The results prove that the bifunctional membrane has excellent anti-corrosion properties. Bifunctional membranes can also be used to prevent the corrosion of metals in other fields.

## 1. Introduction

With the need to reduce traditional fossil fuel usage and environment pollution around the world, the development of clean energy has become extremely important. As a new type of environmentally friendly energy storage device, metal (such as Mg, Zn, Al, etc.)–air batteries have received extensive attention from scientists [1,2]. The improvement in catalytic efficiency has improved the electrochemical performance of metal–air batteries and further broadened their application scope [3,4]. As a special fuel cell, the Al–air battery uses oxygen in the air as the material to activate the cathode and aluminum as the anode reactant. It has the advantages of being lightweight, very safe, and having high specific energy [5,6,7]. Al–air batteries have been widely used in the fields of electric vehicles [8], telecommunications [9], medical equipment [10,11], etc. In order to obtain a high energy density, Al–air batteries mostly use alkaline electrolytes. However, alkaline electrolytes can cause strong hydrogen evolution reactions in the anodes [12]. Due to these serious hydrogen evolution reactions, the actual specific capacity of traditional Al–air batteries is less than 60% of the theoretical value [13]. Therefore, inhibiting the hydrogen evolution reaction of the aluminum anode and increasing the specific capacity of the battery are urgent issues that must be addressed.

The main reason for the hydrogen evolution reaction is that excessive hydroxide ions and water molecules undertake side reactions with the aluminum anode [14]:

(1) $${2\mathrm{Al}+2\mathrm{OH}}^{-}{+6H}_{2}O\to {2\mathrm{Al}(\mathrm{OH})}_{4}^{-}{+3H}_{2}↑$$

To date, scientists have performed a lot of work on inhibiting the attack of hydroxide ions on the aluminum anode. For example, researchers have used an aluminum alloy instead of pure aluminum to increase the overpotential of the hydrogen evolution reaction [15,16], added corrosion inhibitors to the electrolyte to restrict the migration rate of hydroxide ions [17,18], and coated an anti-corrosion film on the surface of the aluminum anode to prevent direct contact between the hydroxide ions and the anode [19,20]. The above methods do inhibit the corrosion of the aluminum anode to a certain extent. However, according to the equation of the side reaction, it is not only the excess hydroxide ions, but also, and more importantly, the excess water molecules that cause hydrogen evolution corrosion. Most current research is aimed at the inhibition of hydroxide ions, and few studies have addressed the restriction of water molecules. 

In relevant anti-corrosion studies in other fields, the importance of inhibiting both water and hydroxide ions simultaneously in order to prevent corrosion has been realized. Cui et al. [21] fabricated a hydrophobic micro-oxidation/zinc stearate composite coating on a Mg alloy through electrodeposition. The hydrophobic coating showed excellent corrosion resistance and could be used for the corrosion protection of mechanical micro-cracks. A hydrophobic Mg(OH)_2_/calcium myristate film was prepared by Zheng et al. [22] to prevent the corrosion of Mg metal parts, and this film could significantly extend the service life of metal parts. However, few studies have been undertaken on bifunctional membranes with both anti-corrosion and hydrophobicity properties for the corrosion protection of the anode in Al–air batteries. In our previous research [23], we prepared a Janus-type membrane with anti-corrosion and hydrophobic functions on the surface of an aluminum anode used in an Al–air battery. The Janus-type membrane showed excellent corrosion resistance, which greatly improved the specific capacity of the Al–air battery. The membrane was composed of two layers of membranes with different functions, and the preparation process was complicated.

Here, we prepare an innovative bifunctional Al_2_O_3_@Polyacrylonitrile (PAN) membrane with both hydrophobic and anti-corrosion functions, which will be used to inhibit the hydrogen evolution reaction. Al_2_O_3_ is a commonly used corrosion inhibitor in Al–air batteries [24,25], and is generally added to an electrolyte to inhibit hydrogen evolution corrosion. PAN is a hydrophobic polymer that is often used in the hydrophobic layer of mask filters [26]. Adding a bifunctional membrane with both hydrophobicity and corrosion resistance properties between the anode and the electrolyte could prevents the invasion into the aluminum anode of excessive hydroxide ions and water molecules simultaneously. 

## 2. Materials and Methods

### 2.1. Materials

The reagents used to prepare the bifunctional membrane, including Al_2_O_3_ nanodispersion (the mass fraction of Al_2_O_3_ is 20 wt. %), PAN, N,N-dimethylformamide (DMF), and KOH flakes, were all purchased from Shanghai Macklin Biochemaical Co., Ltd. (Shanghai, China) Nickel foam and aluminum foil were used as the current collectors and metal anode, respectively. For the air cathode, the activated carbon, ether black, MnO_2_ nanoparticles, and organic solvent N-methyl-2-pyrrolidone (NMP) were purchased from Sinopharm Chemical Reagent Co., Ltd. (Shanghai, China).

### 2.2. Membrane Preparation

**Figure 1:**
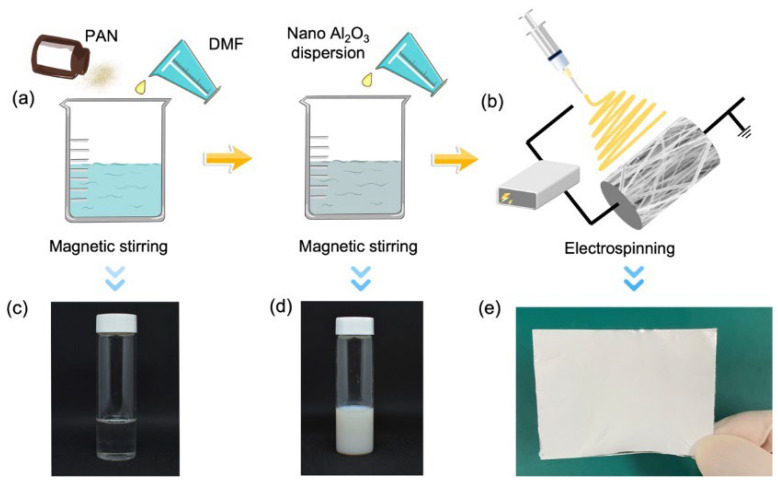
(**a**) The preparation of the precursor solution. (**b**) The electrospinning process of the bifunctional membrane. Photograph of the mixed solution of PAN and DMF (**c**), the precursor solution (**d**), and the bifunctional membrane (**e**).

The preparation of the precursor solution is illustrated in Figure 1a. The PAN was added to the DMF solution, and then heated and stirred at 60 °C for 12 h until the solution was uniform (Figure 1c). Subsequently, the Al_2_O_3_ nanodispersion was added to the above solution, and stirred at 60 °C for 30 min to obtain a uniform white solution as the precursor solution (Figure 1d). In this study, the mass ratios of the Al_2_O_3_ nanodispersion, the PAN and the DMF were (0.5, 0.7, 0.9, 1.1, 1.3):1:10, and the mass fractions of Al_2_O_3_ were 0.77 wt. %, 1.13 wt. %, 01.48 wt. %, 1.82 wt. %, and 2.14 wt. %, respectively. We filled the precursor solution into the syringe pump at a flow rate of 2.0 mL/h for electrospinning. The substrate was aluminum foil, and the distance between the nozzle and the substrate was 14 cm. The voltage was fixed at 12 kV. The electrospinning process is shown in Figure 1b. After electrospinning, the membrane was dried under natural conditions for more than 12 hours, so that the solvent in the membrane could be completely volatilized. Finally, an Al_2_O_3_@PAN bifunctional membrane was formed. A photo of the prepared bifunctional membrane is shown in Figure 1e.

### 2.3. Characterization and Electrochemical Tests

**Figure 2:**
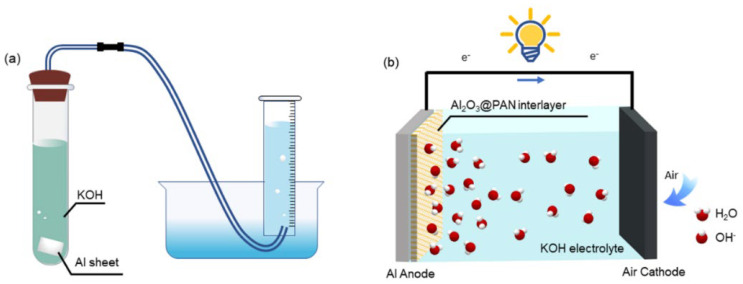
(**a**) Experimental setup of hydrogen evolution test. (**b**) Schematic diagram of the Al−air battery.

The morphological structure of the bifunctional membrane was investigated using a scanning electron microscope (SEM, Su-8010, Hitachi, Tokyo, Japan). The crystal phase of the membrane was analyzed by X-ray diffraction (XRD) (Bruker D8 Advance, Bruker, Karlsruhe, Germany) with a Cu-Kα radiation of 0.1541 nm as the X-ray source. The hydrogen evolution experiment was undertaken in a purpose-built experimental device, as shown in Figure 2a. The aluminum foil with bifunctional membranes on both sides was placed in a 4 M KOH solution, and the hydrogen evolution rate was tested at room temperature. The wettability of the membrane was examined by water contact angle (CA) measurements (DSA30, Kruss, Hamburg, Germany) with 3 μL water droplets. The N_2_ adsorption–desorption isotherms of different membranes were characterized by specific surface area and pore size analysis, using ASAP 2020 (Micromeritics, Norcross, GA, USA).

The electrochemical test was carried out with a three-electrode system on an electrochemical workstation (RST 5000, RST, Suzhou, China). Potentiodynamic polarization was measured at a scan rate of 1 mV/s. Hg/HgO and a platinum wire were used as the reference and counter electrodes, respectively. Electrochemical impedance spectroscopy (EIS) measurements were performed in a frequency range from 100 kHz to 0.01 Hz, and at an AC amplitude of 10 mV. The Al–air batteries were assembled with a sandwich structure; the schematic diagram is shown in Figure 2b. The constant current was discharged using a battery testing system (CT2001A, LAND, Wuhan, China).

## 3. Results

### 3.1. Characterization of the Al_2_O_3_@PAN Membrane

**Figure 3:**
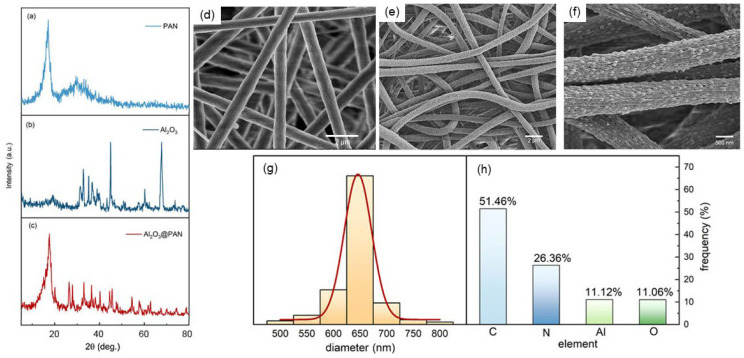
The diffraction spectra of the PAN (**a**), Al_2_O_3_ (**b**), and Al_2_O_3_@PAN (**c**). (**d**) SEM micrograph of the pure PAN nanofibers. (**e**,**f**) SEM micrograph of the Al_2_O_3_@PAN nanofibers. (**g**) The diameter distribution of the Al_2_O_3_@PAN nanofibers. (**h**) The ratio of each element in the nanofibers.

Figure 3a–c show the XRD patterns of the pure PAN membrane, the pure Al_2_O_3_ powder, and the Al_2_O_3_@PAN nanofibers. For the pure PAN, the peaks appeared at 17.2° and 30°, which is consistent with the research of Sabetzadeh et al. [27]. For the pure Al_2_O_3_, the diffraction peaks appeared at 37.6°, 39.5°, 45.8°, 60.9°, 67.1°, and 76.8°, and can be assigned to the (3 1 1), (2 2 2), (4 0 0), (5 1 1), (4 4 0) and (6 2 0) planes of Al_2_O_3_, respectively. The locations of the peaks adhere to the standard JCPDS (79-1558) [28]. When the PAN was mixed with Al_2_O_3_, the characteristic peaks of the two did not change significantly, but their sizes were reduced. This implies that Al_2_O_3_ was successfully embedded in the polymer, reducing the crystallinity of the PAN. Similar results were also reported by Pendy et al. [29]. Figure 3d shows the SEM results of the pure PAN nanofibers. The nanofibers are smooth and flat and have a uniform size. Figure 3e,f shows the SEM results of the composite membrane prepared by electrospinning. The diameter of the nanofibers is about 650 nm, and the diameter distribution is shown in Figure 3g. From the detailed view in Figure 3f, we can see that a large number of nanoparticles are distributed on the surface of the fiber. The Al_2_O_3_ nanoparticles are securely attached to the surfaces of the nanofibers. Figure 3h shows the elemental contents of C, N, Al, and O.

**Figure 4:**
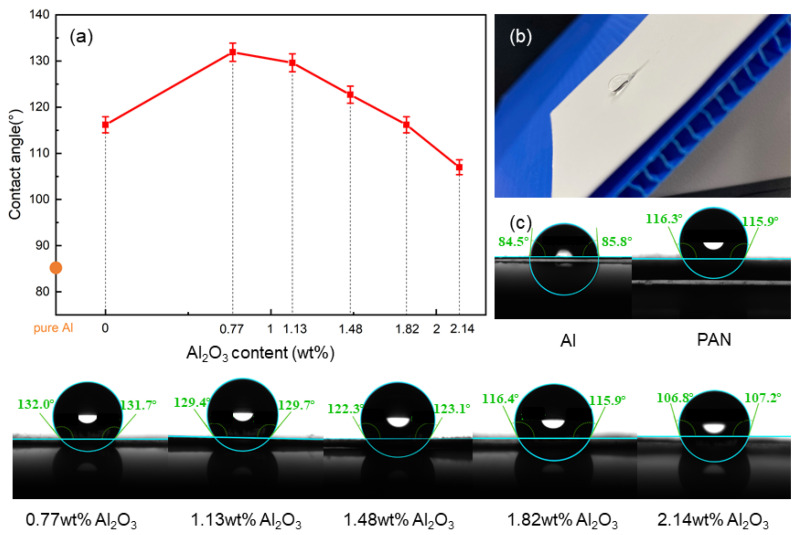
(**a**) Contact angle of the Al anode coated with different membranes. (**b**) Photograph of a droplet on the surface of the anti-corrosion membrane. (**c**) Photographs of the contact angle.

Surface wettability is an important factor affecting corrosion resistance. In this study, the functional membrane was prepared by electrospinning it onto the surface of the aluminum anode. Figure 4a shows the surface wettability values of the aluminum anodes with different coatings. It can be seen from the figure that the surface of pure aluminum is hydrophilic, and its contact angle is about 85°. After adding the pure PAN membrane to the surface of pure aluminum, it gained obvious hydrophobicity, and the contact angle became about 116°, mainly due to the high hydrophobicity of PAN. When the membrane was supplemented with a small amount (0.77 wt. %) of Al_2_O_3_, the contact angle reached 133°. The morphology of the droplet on the surface of the membrane is shown in Figure 4b. As the Al_2_O_3_ content in the composite membrane increased, the contact angle gradually decreased. This may be due to the fact that Al_2_O_3_ nanoparticles were embedded in the PAN nanofibers, which increased the roughness of the membrane and made the penetration of water easier.

**Figure 5:**
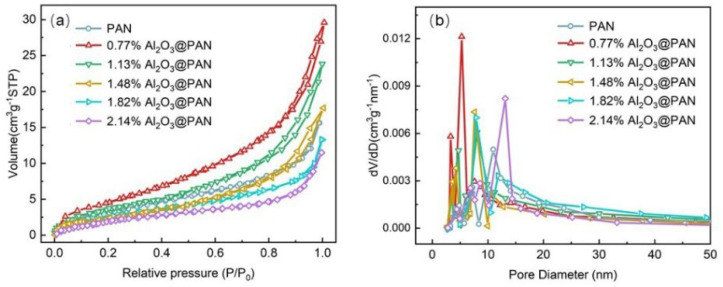
(**a**) BET surface area and (**b**) pore size distribution of different membranes.

The specific surface area is here obtained using the N_2_ adsorption–desorption isotherms, which are calculated according to the Brunner–Emmet–Teller (BET) model. The results are shown in Figure 5a. The specific surface area of the pure PAN membrane was 8.57 cm^3^/g, and this increased after adding a small amount of Al_2_O_3_. As the Al_2_O_3_ content increased, the specific surface area of the membrane decreased. The pore size distribution has been determined based on the Barrett–Joyner–Halenda (BJH) test, and the results are shown in Figure 5b. It can be seen from the figure that the pore size distributions of different membranes were homogeneous. The pore size of the pure PAN membrane was larger. When a small amount of Al_2_O_3_ was added to the membrane, the pore size of the membrane was significantly reduced. The pore size of the membrane did not change significantly when the Al_2_O_3_ content was 1.13–1.82 wt. %. However, as the Al_2_O_3_ content continued to increase, the pore size increased significantly. The surface areas and most common pore sizes of different samples are listed in Table 1.

**Figure 6:**
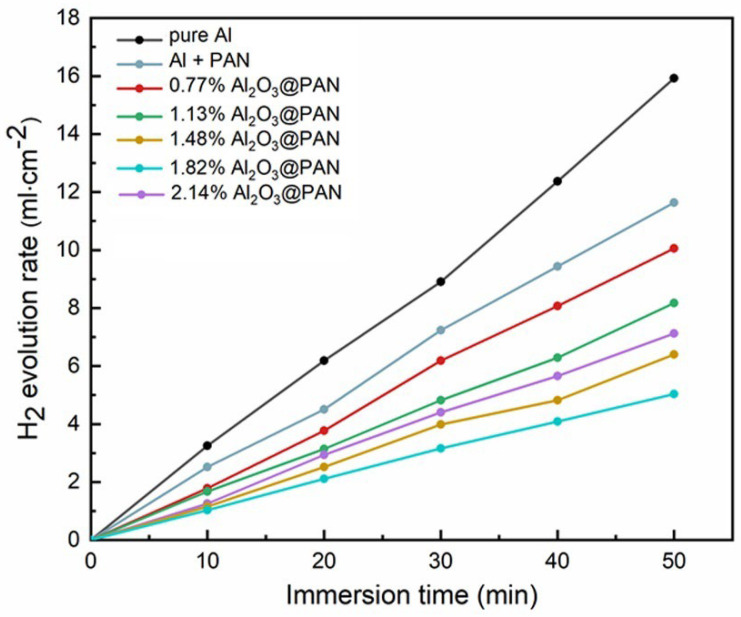
Hydrogen evolution rates of the Al anodes coated with different anti-corrosion membranes in 4 M KOH solutions.

The aluminum anode with a bifunctional membrane was placed in a 4 M KOH solution, and the amount of hydrogen precipitated by corrosion was tested via the air exhaust method. Figure 6 shows the curve of the hydrogen evolution rate in the aluminum anodes. Compared with the pure aluminum anode, the hydrogen evolution rate of the aluminum anode with the functional membrane was significantly reduced. The pure PAN hydrophobic membrane somewhat suppressed the hydrogen evolution corrosion rate. When Al_2_O_3_ was added to the membrane, the hydrogen evolution rate was greatly reduced. When the Al_2_O_3_ content increased from 0.77 to 1.82 wt. %, the hydrogen evolution rate decreased accordingly. In an alkaline solution, Al_2_O_3_ can react with OH^−^ to form (AlO_2_)^−^, which will consume part of the OH^−^ and thus reduce its concentration near the surface of the aluminum anode [30,31]. Therefore, although the hydrophobicity of the membrane decreased and the pore size increased, the addition of Al_2_O_3_ effectively reduced the level of excess OH^−^ near the aluminum anode. It is worth noting that when the Al_2_O_3_ content in the membrane increased to 2.14 wt. %, the hydrogen evolution rate increased. Excessive hydroxide ions became attached to the PAN fibers, and the pore sizes of the membranes thus increased significantly. Under these conditions, too many water molecules, and possibly even hydroxide ions, can penetrate the membrane and reach the surface of the aluminum anode, which will result in a reduction in the corrosion inhibition performance of the membrane.

### 3.2. Corrosion Resistance of the Al_2_O_3_@PAN Membrane

**Figure 7:**
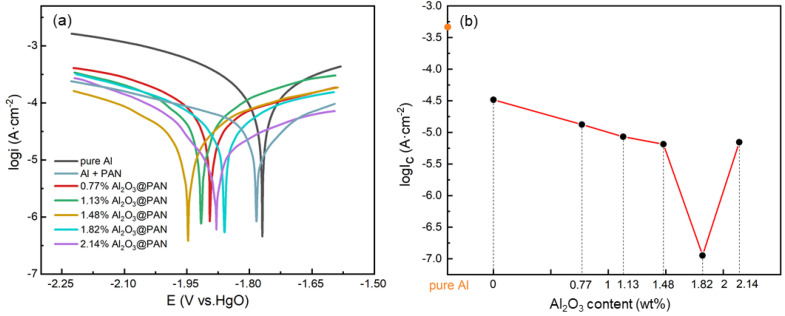
(**a**) Polarization curves of different Al anodes in 4 M KOH solution. (**b**) The relationship between corrosion current and Al_2_O_3_ content in anti-corrosion membranes.

Figure 7a shows the potentiodynamic polarization curves of aluminum electrodes with different functional membranes in a 4 M KOH solution. The corrosion reactions of aluminum in alkaline solutions include cathodic and anodic processes, as described in Equations (2) and (3) [32,33]:

(2) $${2H}_{2}{O+2e}^{-}\to {2\mathrm{OH}}^{-}{+H}_{2}↑$$

(3) $${\mathrm{Al}+4\mathrm{OH}}^{-}\to {\mathrm{Al}(\mathrm{OH})}_{4}^{-}{+3e}^{-}$$

The corresponding corrosion potential ($E_{coor}$) and corrosion current density ($I_{coor}$) obtained by fitting the curves are listed in Table 2. The Tafel curve shows that the corrosion potential drops after adding the corrosion-resistant membrane. The $E_{coor}$ of pure aluminum was −1.77 V, the $E_{coor}$ of the aluminum electrode with a pure PAN membrane was −1.79 V, and the $E_{coor}$ of the aluminum electrode with a bifunctional membrane (1.48 wt. % Al_2_O_3_@PAN) reached −1.95 V. In the aluminum electrode with a 2.14 wt. % Al_2_O_3_@PAN membrane, the $E_{coor}$ slightly decreased to −1.87 V. This explains why the addition of a functional membrane increases the overpotential of the hydrogen evolution corrosion of the aluminum electrode. Figure 7b shows the dependence of $I_{coor}$ on Al_2_O_3_ content. The polarization curve of the aluminum electrode with a pure PAN membrane is suppressed significantly, especially in the cathodic part, indicating that PAN acted as a cathodic inhibitor, and mainly affected the efficiency of the cathodic reaction. When the content of Al_2_O_3_ increased from 0.77 to 1.82 wt. %, the $I_{coor}$ decreased from 8.23 to 3.23 mA/cm^2^. The negative shift in the corrosion voltage and the lower corrosion current indicate that the bifunctional membrane suppressed both the cathodic and the anodic process. When the Al_2_O_3_ increased to 2.14 wt. %, the $I_{coor}$ increased to 6.41 mA/cm^2^. The main reason for this phenomenon is that more Al_2_O_3_ nanoparticles were attached to the PAN nanofibers in the 2.14 wt. % Al_2_O_3_@PAN membrane. As a result, the hydrophobicity of the membrane decreased, and the inhibitory effect against water molecules was limited, resulting in a reduced ability to suppress the hydrogen evolution corrosion of the membrane. This result is consistent with the change in the $E_{coor}$, and is corroborated by the conclusion of the hydrogen evolution experiment. The corrosion inhibition rate can be calculated by the following equation [34]:

(4) $$\eta \%=\frac{I_{coor}-I_{coor}^{\prime }}{I_{coor}}\times 100$$

The corrosion inhibition rate of the aluminum electrode with a pure PAN membrane was 69.9%, and that of aluminum with a 1.82 wt. % Al_2_O_3_@PAN membrane reached the highest value of 89.24%.

**Figure 8:**
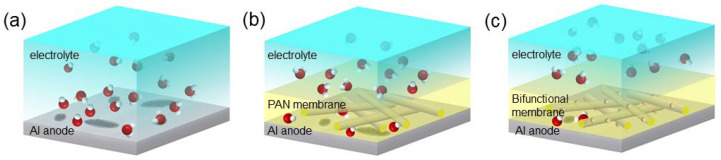
Schematic diagram of the anti-corrosion mechanism of the bifunctional membrane. (**a**) Traditional Al anode without any surface modification. (**b**) The Al anode with a hydrophobic PAN membrane. (**c**) The Al anode coated with a bifunctional membrane.

Situated between the electrode and the electrolyte, the functional membrane can effectively prevent the direct contact of the aluminum electrode and the electrolyte. The surface of a traditional pure aluminum anode is not modified, and the corrosion damage it permits is thus serious, as shown in Figure 8a. A PAN membrane with hydrophobic properties can significantly limit the invasion of water molecules, and has a certain corrosion inhibition effect, as shown in Figure 8b. A bifunctional membrane can not only inhibit the invasion of hydroxide ions, but can also block excess water molecules, as shown in Figure 8c. Through the above polarization curve analysis, we found that the two components (PAN and Al_2_O_3_) are indispensable to a bifunctional film. The experimental results show that when the membrane only contains PAN, the corrosion reaction efficiency of the cathodic part can be reduced. After the addition of Al_2_O_3_, the corrosion inhibition rate was significantly improved, indicating that Al_2_O_3_ effectively hindered hydroxide ion entry. In the study of Lee et al. [35], Al_2_O_3_ was adhered to the surface of a zinc electrode to inhibit its hydrogen evolution corrosion in an alkaline solution. Here, after adding PAN to the anti-corrosion membrane, we achieved a higher corrosion inhibition rate due to the hydrophobic property of PAN.

**Figure 9:**
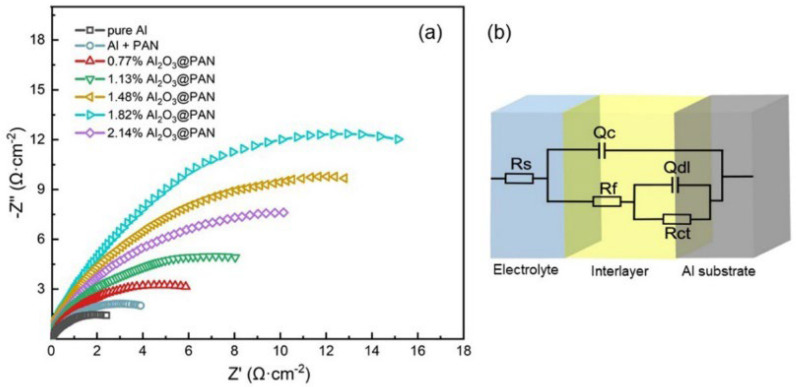
(**a**) Electrochemical impedance spectra in Nyquist plots of different Al anodes. (**b**) Equivalent circuits used for fitting the EIS.

The electrochemical behavior of an aluminum electrode with a protective membrane in an alkaline electrolyte was further tested by EIS, in order to obtain more detailed information on the mechanisms and the electrode membrane’s structure [36]. Figure 9a shows the Nyquist curve of the aluminum anode in 4 M KOH. The impedance spectrum of the aluminum electrode in an alkaline solution shows characteristics of capacitance, indicating that the charge transfer resistance is dominant in the corrosion process [37,38]. Figure 9b shows an equivalent circuit diagram, which is used to fit EIS. In the equivalent circuit, $R_{s}$ is the solution resistance, $R_{f}$ and $Q_{c}$ are the resistance and capacitance of the protective membrane, respectively, $R_{ct}$ is the charge transfer resistance, and $Q_{dl}$ is the electric double layer capacitance. The impedance parameters by fitting the EIS can be seen in Table 3. According to the equivalent circuit, the values of $R_{f}$ and $Q_{c}$ express the characteristics of the functional membrane on the surface of the aluminum electrode. The $R_{f}$ of the aluminum electrode with a Al_2_O_3_@PAN membrane is significantly greater than that of the single-functional PAN membrane, indicating that the coexistence of Al_2_O_3_ nanoparticles and PAN increases the membrane’s resistance and makes it more protective. When the content of Al_2_O_3_ ranges 0.77 to 1.82 wt. %, as the Al_2_O_3_ fraction increases, the $R_{f}$ value of the composite membrane increases. When the Al_2_O_3_ content reaches 2.14 wt. %, due to the reduced hydrophobicity of the membrane, more water molecules can pass through (see the previous discussion of hydrophobicity), and the $R_{f}$ value of the composite membrane will decrease. The charge transfer resistance accurately reflects the protective effect of the functional membrane on the electrode. After adding a functional membrane onto the surface of the aluminum anode, the $R_{ct}$ value became significantly higher than that of the pure aluminum anode. The $R_{ct}$ of the aluminum anode with a bifunctional membrane showed clear improvements. This proves that the Al_2_O_3_@PAN membrane has the best protective effect on an aluminum electrode by effectively hindering the erosion effect of the large number of hydroxide ions and water molecules on the aluminum surface, and its anti-corrosion effect is better than that of a PAN membrane with a single hydrophobic function. The $R_{ct}$ values of the aluminum electrodes with bifunctional membranes rank in the following order: 0.77 wt. % Al_2_O_3_@PAN < 1.13 wt. % Al_2_O_3_@PAN < 1.48 wt. % Al_2_O_3_@PAN < 2.14 wt. % Al_2_O_3_@PAN < 1.82 wt. % Al_2_O_3_@PAN. Its greater charge transfer resistance proves that the composite Al_2_O_3_@PAN membrane can effectively reduce the size of the reaction sites [39] on the surface of the aluminum anode. The $R_{ct}$ value of 1.82 wt. % Al_2_O_3_@PAN is the largest, indicating that its corrosion effect is the best, which is consistent with the results of the polarization test.

**Figure 10:**
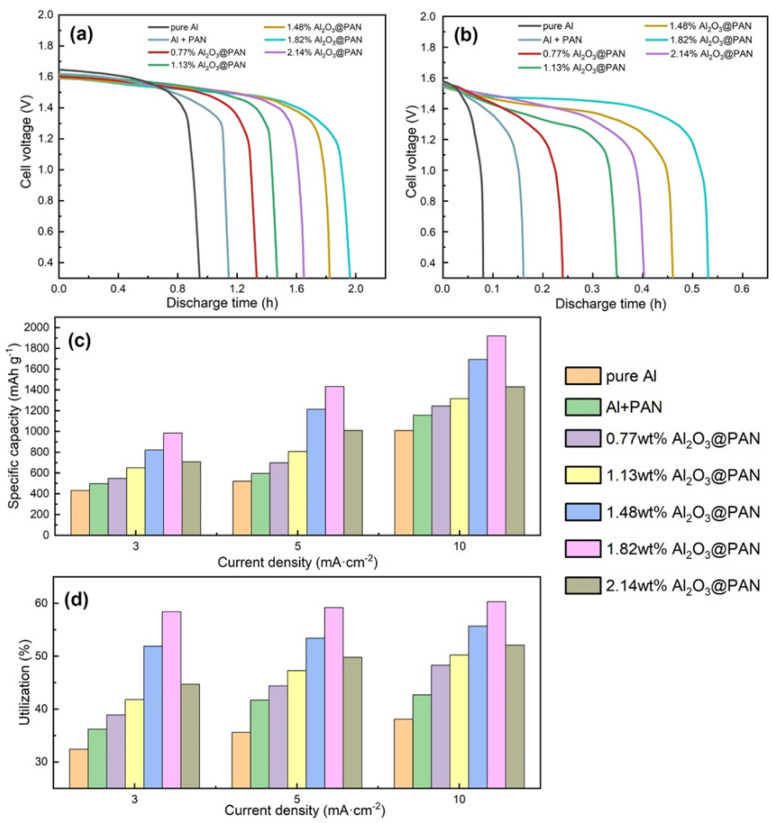
Discharge curves of Al–air batteries with different Al anodes at 3 (**a**) and 10 mA/cm^−2^ (**b**). The specific capacities (**c**) and utilizations (**d**) of different Al anodes.

Considering the excellent anti-corrosion properties of the bifunctional membrane, we placed it between the aluminum electrode and the electrolyte to protect the anode from self-corrosion. Figure 10a,b show the discharge performance of the assembled Al–air batteries at 3 and 10 mA/cm^−2^. It can be seen from the figure that after adding an anti-corrosion membrane, the discharge time of the battery was significantly prolonged—most notably that of the battery with a bifunctional anti-corrosion membrane. It is worth noting that even when a bifunctional anti-corrosion membrane is added to the battery, its discharge voltage platform remains very similar to that of a battery with a pure aluminum anode. This proves that when a bifunctional membrane is added, the supply of hydroxide ions can still meet the normal discharge requirements of the battery. When the content of Al_2_O_3_ in the bifunctional membrane is 1.82 wt. %, the discharge time of the Al–air battery is the longest, indicating that the corrosion inhibition effect of the 1.82 wt. % Al_2_O_3_@PAN composite membrane is the best.

Figure 10c,d shows the specific capacity and the utilization of Al–air batteries with different membranes, and Table 4 summarizes the values. The anode utilization rate of the Al–air battery is determined by the following equation [40,41]:

(5) $$U_{\alpha }\%=100\times \frac{9It}{∆mF}$$

where I represents the current (in A), t is the discharge time (in h), ∆m is the weight loss (in g) and F is the Faraday constant. It can be seen from Figure 10c that the bifunctional membrane increases the specific capacity of Al–air batteries effectively. The specific capacity of the Al–air battery with a 1.82 wt. % Al_2_O_3_@PAN membrane reached 1950 mAh g^−1^ at a current density of 10 mA cm^−2^. This specific capacity offers clear advantages compared to a traditional Al–air battery with liquid electrolyte. Compared with previous studies [17], this research has obtained higher specific capacities under the same discharge current density. Figure 10d compares the anode utilizations of Al–air batteries with different membranes. The results are consistent with those of the Tafel, EIS and constant current discharge tests. The utilization rate of an aluminum anode with a bifunctional 1.82 wt. % Al_2_O_3_@PAN membrane was the highest, at 61.2%. The anode utilization rate obtained when using a bifunctional membrane is comparable to that achieved by organic–inorganic inhibitors [17]. This proves that the bifunctional membrane effectively reduces the degree of unnecessary consumption displayed by the aluminum electrode due to self-corrosion. Adding the bifunctional membrane can significantly increase the anode utilization rate of the Al–air battery, which helps to reduce the waste of aluminum resources, and is thus of great significance to environmental protection and energy saving.

## 4. Conclusions

In this study, a bifunctional anti-corrosion membrane for Al–air batteries was prepared by electrospinning. The components of the bifunctional membrane are PAN and Al_2_O_3_ nanoparticles. The PAN displays excellent hydrophobicity, and Al_2_O_3_ nanoparticles can prevent the attack of hydroxide ions on the aluminum anode. Our composite membrane is placed between the aluminum electrode and the electrolyte, which can effectively inhibit self-corrosion. Using it in Al–air batteries can significantly improve the anode utilization and specific capacity. We found that a higher corrosion inhibition rate can be obtained by increasing the Al_2_O_3_ content in the bifunctional membrane appropriately, but too many Al_2_O_3_ nanoparticles will affect the hydrophobicity of the membrane, resulting in a decrease in the corrosion inhibition rate. The corrosion inhibition rate of the 1.82 wt. % Al_2_O_3_@PAN membrane reached 89.24% in an alkaline electrolyte, while the specific capacity was 1950 mAh g^−1^ and the utilization rate of the aluminum anode reached 61.2%. The bifunctional membrane proposed in this research has a low cost, significant effectiveness, simple preparation requirements, and a minimal impact on the discharge of the battery. The membrane can effectively inhibit the hydrogen evolution reaction of the aluminum anode, increase the specific capacity, improve the utilization rate of the aluminum anode, and reduce the waste of aluminum resources. The results of this study offer a new approach to metal corrosion inhibition under alkaline conditions.

## Figures and Tables

**Table 1:** Pore characteristics of different samples.

Samples	Specific Surface Area (m^2^/g)	Average Pore Size (nm)
PAN	8.57	11.43
0.77 wt. % Al_2_O_3_@PAN	14.95	6.92
1.13 wt. % Al_2_O_3_@PAN	10.72	7.37
1.48 wt. % Al_2_O_3_@PAN	9.29	7.52
1.82 wt. % Al_2_O_3_@PAN	8.37	7.28
2.14 wt. % Al_2_O_3_@PAN	6.35	13.4

**Table 2:** Corrosion parameters.

Materials	$E_{coor}\;(V)$	$I_{coor}\;(\mathrm{mA}/{\mathrm{cm}}^{2})$	η%
Pure Al	−1.77	36.88	—
Al with PAN	−1.79	11.10	69.9
Al with 0.77 wt. % Al_2_O_3_@PAN	−1.89	8.23	77.68
Al with 1.13 wt. % Al_2_O_3_@PAN	−1.92	6.10	83.46
Al with 1.48 wt. % Al_2_O_3_@PAN	−1.95	5.52	85.03
Al with 1.82 wt. % Al_2_O_3_@PAN	−1.86	3.23	89.24
Al with 2.14 wt. % Al_2_O_3_@PAN	−1.87	6.41	82.62

**Table 3:** Impedance parameters of different Al anodes obtained by fitting the EIS.

Materials	R_s_ (Ω cm^2^)	R_f_ (Ω cm^2^)	Q_c_ (×10^−4^ μF cm^−2^)	R_ct_ (Ω cm^2^)	Q_dl_ (×10^−2^ μF cm^−2^)
Pure Al	0.23	1.21	0.32	3.13	1.49
Al with PAN	0.24	1.49	2.54	4.94	3.28
Al with 0.77 wt. % Al_2_O_3_@PAN	0.22	1.53	2.43	8.65	4.39
Al with 1.13 wt. % Al_2_O_3_@PAN	0.20	1.67	2.21	12.21	5.16
Al with 1.48 wt. % Al_2_O_3_@PAN	0.21	1.74	2.15	22.32	5.79
Al with 1.82 wt. % Al_2_O_3_@PAN	0.22	1.98	2.06	26.13	6.87
Al with 2.14 wt. % Al_2_O_3_@PAN	0.22	1.70	2.17	17.45	5.38

**Table 4:** The specific capacities and the utilizations of different Al anodes.

Materials	3 (mA cm^−2^)		5 (mA cm^−2^)		10 (mA cm^−2^)	
	Capacities (mAh g^−1^)	Utilization Rate (%)	Capacities (mAh g^−1^)	Utilization Rate (%)	Capacities (mAh g^−1^)	Utilization Rate (%)
Pure Al	432	32.4	521	35.6	1008	38.1
Al with PAN	497	36.2	595	41.7	1156	42.7
Al with 0.77 wt. % Al_2_O_3_@PAN	548	38.9	699	44.4	1245	48.3
Al with 1.13 wt. % Al_2_O_3_@PAN	651	41.8	807	47.3	1316	50.2
Al with 1.48 wt. % Al_2_O_3_@PAN	821	51.9	1214	53.4	1694	55.7
Al with 1.82 wt. % Al_2_O_3_@PAN	984	58.4	1433	59.2	1950	61.2
Al with 2.14 wt. % Al_2_O_3_@PAN	708	44.7	1009	49.8	1430	52.1

## Data Availability

The data presented in this study are available on request from the corresponding author.

## References

[1] Logeshwaran N., Ramakrishnan S., Chandrasekaran S.S., Vinothkannan M., Kim A.R., Sengodan S., Velusamy D.B., Varadhan P., He J.H., Yoo D.J. (2021). An efficient and durable trifunctional electrocatalyst for zinc–air batteries driven overall water splitting. Appl. Catal. B Environ..

[2] Ramakrishnan S., Velusamy D.B., Sengodan S., Nagaraju G., Kim D.H., Kim A.R., Yoo D.J. (2022). Rational design of multifunctional electrocatalyst: An approach towards efficient overall water splitting and rechargeable flexible solid-state zinc–air battery. Appl. Catal. B Environ..

[3] Sathiskumar C., Ramakrishnan S., Vinothkannan M., Kim A.R., Karthikeyan S., Yoo D.J. (2020). Nitrogen-doped porous carbon derived from biomass used as trifunctional electrocatalyst toward oxygen reduction, oxygen evolution and hydrogen evolution reactions. Nanomaterials.

[4] Elayappan V., Shanmugam R., Chinnusamy S., Yoo D.J., Mayakrishnan G., Kim K., Noh H.S., Kim M.K., Lee H. (2020). Three-dimensional bimetal TMO supported carbon based electrocatalyst developed via dry synthesis for hydrogen and oxygen evolution. Appl. Surf. Sci..

[5] Goel P., Dobhal D., Sharma R.C. (2020). Aluminum–air batteries: A viability review. J. Energy Storage.

[6] Luo Z., Yin L., Xiang L., Liu T.X., Song Z., Li Y., Zhou L., Luo K., Wu K., Jiang J. (2021). AuPt Nanoparticles/ Multi-Walled carbon nanotubes catalyst as high active and stable oxygen reduction catalyst for Al–air batteries. Appl. Surf. Sci..

[7] Wei Y., Shi Y., Chen Y., Xiao C., Ding S. (2021). Development of solid electrolytes in Zn-air and Al–air batteries: From material selection to performance improvement strategies. J. Mater. Chem. A.

[8] Elia G.A., Marquardt K., Hoeppner K., Fantini S., Lin R., Knipping E., Peters W., Drillet J.F., Passerini S., Hahn R. (2016). An Overview and Future Perspectives of Aluminum Batteries. Adv. Mater..

[9] Buckingham R., Asset T., Atanassov P. (2021). Aluminum-air batteries: A review of alloys, electrolytes and design. J. Power Sources.

[10] Hu Y., Sun D., Luo B., Wang L. (2019). Recent Progress and Future Trends of Aluminum Batteries. Energy Technol..

[11] Wu P., Wu S., Sun D., Tang Y., Wang H. (2021). A Review of Al Alloy Anodes for Al–Air Batteries in Neutral and Alkaline Aqueous Electrolytes. Acta Metall. Sin. English Lett..

[12] Li X., Li J., Zhang D., Gao L., Qu J., Lin T. (2021). Synergistic effect of 8-aminoquinoline and ZnO as hybrid additives in alkaline electrolyte for Al–air battery. J. Mol. Liq..

[13] Chen X., Ali I., Song L., Song P., Zhang Y., Maria S., Nazmus S., Yang W., Dhakal H.N., Li H. (2020). A review on recent advancement of nano-structured-fiber-based metAl–air batteries and future perspective. Renew. Sustain. Energy Rev..

[14] Wu S., Zhang Q., Sun D., Luan J., Shi H., Hu S., Tang Y., Wang H. (2020). Understanding the synergistic effect of alkyl polyglucoside and potassium stannate as advanced hybrid corrosion inhibitor for alkaline aluminum-air battery. Chem. Eng. J..

[15] Wu Z., Zhang H., Zou J., Shen X., Qin K., Ban C., Cui J., Nagaumi H. (2020). Enhancement of the discharge performance of Al-0.5Mg-0.1Sn-0.05Ga (wt. %) anode for Al–air battery by directional solidification technique and subsequent rolling process. J. Alloys Compd..

[16] Ren J., Ma J., Zhang J., Fu C., Sun B. (2019). Electrochemical performance of pure Al, Al–Sn, Al–Mg and Al–Mg–Sn anodes for Al–air batteries. J. Alloys Compd..

[17] Jiang H., Yu S., Li W., Yang Y., Yang L., Zhang Z. (2020). Inhibition effect and mechanism of inorganic-organic hybrid additives on three-dimension porous aluminum foam in alkaline Al–air battery. J. Power Sources.

[18] Kang Q.X., Zhang T.Y., Wang X., Wang Y., Zhang X.Y. (2019). Effect of cerium acetate and L-glutamic acid as hybrid electrolyte additives on the performance of Al–air battery. J. Power Sources.

[19] Mutlu R.N., Yazıcı B. (2019). Copper-deposited aluminum anode for aluminum-air battery. J. Solid State Electrochem..

[20] Lee J., Yim C., Lee D.W., Park S.S. (2017). Manufacturing and characterization of physically modified aluminum anodes based air battery with electrolyte circulation. Int. J. Precis. Eng. Manuf. Green Technol..

[21] Cui L., Liu H., Zhang W., Han Z., Deng M. (2017). Journal of Materials Science & Technology Corrosion resistance of a superhydrophobic micro-arc oxidation coating on Mg-4Li-1Ca alloy. J. Manuf. Syst..

[22] Zheng Z., Wei Y., Jing Z., Rong X., Zeng C., Qin F., Zhen W., Wang L. (2021). Corrosion Resistance of Superhydrophobic Mg(OH)2/Calcium Myristate Composite Coating on Magnesium Alloy AZ31. Acta Metall. Sin. English Lett..

[23] Wang Y., Yu Y., Wang J., Peng L., Zuo Y., Zuo C. (2021). Novel Multifunctional Janus-Type Membrane on Al Anode for Corrosion Protection. Adv. Mater. Interfaces.

[24] Yu Y., Zuo Y., Zhang Z., Wu L., Ning C., Zuo C. (2019). Al2O3 Coatings on Zinc for Anti-Corrosion in Alkaline Solution by Electrospinning. Coatings.

[25] Ehsani A., Mahjani M.G., Nasseri M., Jafarian M. (2014). Influence of electrosynthesis conditions and Al2O3 nanoparticles on corrosion protection effect of polypyrrole films. Anti-Corrosion Methods Mater..

[26] Wang J., Hou L., Yao Z., Dou W., Li G., Zhang L. (2021). Antifouling sandwich-structured electrospun nanofibrous membranes by integrating fluffy and hydrophobic layers for long-term airborne particulate matter segregation. Environ. Sci. Nano.

[27] Sabetzadeh N., Akbar A., Javanbakht M. (2018). Porous PAN micro/nano fi ber separators for enhanced lithium-ion battery performance. Solid State Ionics.

[28] Bahari B.S.M.B.A. (2017). Studying electrical characteristics of Al2O3/PVP nano-hybrid composites as OFET gate dielectric. J. Mater. Sci. Mater. Electron..

[29] Pandey M., Joshi G.M., Mukherjee A., Thomas P. (2016). Electrical properties and thermal degradation of poly(vinyl chloride)/polyvinylidene fluoride/ZnO polymer nanocomposites. Polym. Int..

[30] Wongrujipairoj K., Poolnapol L., Arpornwichanop A., Suren S., Kheawhom S. (2017). Suppression of zinc anode corrosion for printed flexible zinc-air battery. Phys. Status Solidi B.

[31] Mori R. (2014). A novel aluminium-Air rechargeable battery with Al2O3 as the buffer to suppress byproduct accumulation directly onto an aluminium anode and air cathode. RSC Adv..

[32] Verma C.B., Singh P., Bahadur I., Ebenso E.E., Quraishi M.A. (2015). Electrochemical, thermodynamic, surface and theoretical investigation of 2-aminobenzene-1,3-dicarbonitriles as green corrosion inhibitor for aluminum in 0.5 M NaOH. J. Mol. Liq..

[33] Nestoridi M., Pletcher D., Wood R.J.K., Wang S., Jones R.L., Stokes K.R., Wilcock I. (2008). The study of aluminium anodes for high power density Al/air batteries with brine electrolytes. J. Power Sources.

[34] Jo Y.N., Kang S.H., Prasanna K., Eom S.W., Lee C.W. (2017). Shield effect of polyaniline between zinc active material and aqueous electrolyte in zinc-air batteries. Appl. Surf. Sci..

[35] Lee S.M., Kim Y.J., Eom S.W., Choi N.S., Kim K.W., Cho S.B. (2013). Improvement in self-discharge of Zn anode by applying surface modification for Zn-air batteries with high energy density. J. Power Sources.

[36] Fan L., Lu H., Leng J. (2015). Performance of fine structured aluminum anodes in neutral and alkaline electrolytes for Al–air batteries. Electrochim. Acta.

[37] Fan H., Li S., Zhao Z., Wang H., Shi Z., Zhang L. (2011). Inhibition of brass corrosion in sodium chloride solutions by self-assembled silane films. Corros. Sci..

[38] Emregül K.C., Atakol O. (2003). Corrosion inhibition of mild steel with Schiff base compounds in 1 M HCl. Mater. Chem. Phys..

[39] Ma X., Xu L., Wang W., Lin Z., Li X. (2017). Synthesis and characterisation of composite nanoparticles of mesoporous silica loaded with inhibitor for corrosion protection of Cu-Zn alloy. Corros. Sci..

[40] Zhu C., Yang H., Wu A., Zhang D., Gao L., Lin T. (2019). Modified alkaline electrolyte with 8-hydroxyquinoline and ZnO complex additives to improve Al–air battery. J. Power Sources.

[41] Wang Q., Miao H., Xue Y., Sun S., Li S., Liu Z. (2017). Performances of an Al-0.15 Bi-0.15 Pb-0.035 Ga alloy as an anode for Al–air batteries in neutral and alkaline electrolytes. RSC Adv..

